# Serum exosomal microRNA profiling reveals a down-regulation of hsa-miR-124-3p in patients with severe acne

**DOI:** 10.3389/fimmu.2025.1554811

**Published:** 2025-06-23

**Authors:** Shujuan Zhang, Yimin Liang, Manqi Xia, Xin Tian, Ziyan Chen, Ling Lin, Jingyao Liang, Yumei Liu

**Affiliations:** ^1^ Institute of Dermatology, Guangzhou Medical University, Department of Dermatology, Guangzhou Dermatology Hospital, Guangzhou, China; ^2^ Department of Dermatology, Guangzhou Twelfth People’s Hospital, Guangzhou, China

**Keywords:** severe acne, serum exosome, miR-124-3p, differential expression, target genes

## Abstract

**Background:**

Acne is a chronic inflammatory skin disease affecting pilosebaceous unit. However, its specific mechanism remain incompletely understood.

**Objectives:**

This study aims to identify and analyze the differential expression of serum exosomal miRNA in severe acne, revealing new insights into the pathogenesis of acne.

**Methods:**

MiRNAs were extracted from serum exosomes of 15 patients with severe acne and 15 healthy controls. MiRNA libraries were constructed and sequenced using Illumina HiSeq 2500. The DESeq2R was applied to identify differentially expressed miRNAs. The candidate target genes were predicted using multiple miRNA databases. The DAVID database was used to enrich GO function and KEGG pathway analysis of target genes. Cytoscape3.10.0 was employed to construct a PPI interaction network and further screen hub genes. The most significantly differentially expressed miRNAs were validated using RT-qPCR detection.

**Results:**

Small RNA-Seq analysis identified a total of 96 serum exosome miRNAs, with 33 up-regulated and 63 down-regulated. Target prediction across four miRNA databases identified 10,569 target genes. GO analysis showed that target genes were mainly enriched in transcriptional regulation, signal transduction and protein binding; KEGG analysis revealed enrichment in 160 pathways including PI3K-Akt and MAPK signaling pathway. Cytoscape 3.10.0 identified 7 hub genes: PIK3R1, PIK3CA, SRC, EGFR, JAK2, ERBB2, and IGF1R, along with 35 corresponding differentially expressed miRNAs. RT-qPCR results indicated a significant reduction in exosomal miR-124-3p levels in severe acne.

**Conclusions:**

Serum exosomal miRNA expression in patients with severe acne significantly differed from that in healthy individuals. The exosomal miR-124-3p expression was markedly reduced in severe acne compared to healthy controls. Consequently, the increase of miR-124-3p expression may have potential therapeutic implications for severe acne.

## Introduction

Acne is a chronic inflammatory skin disease associated with human sebaceous glands, predominantly affecting adolescents (1). The inflammatory lesions manifest as papules, pustules, nodules or cysts, potentially resulting in lifelong scarring and psychological discomfort (2). It is currently believed that acne is associated with androgen-induced increased sebum production, abnormal keratinisation, bacterial colonization of Propionibacterium acnes(*P.acnes*), and inflammation (3). However the exact pathogenesis remains unclear.

Exosomes are extracellular vesicles that transport various molecules, including DNA, RNA, proteins, and lipids, outside the cell (4). MicroRNAs (miRNAs), a type of non-coding RNAs, play crucial roles in post-transcriptional gene regulation by binding to the 3’-untranslated region(UTR) of messenger RNAs (mRNAs) and reducing or inhibiting translation (5). MiRNAs are essential to numerous biological processes, including cell proliferation (6), differentiation (7), and apoptosis (8). Human miRNAs are genomically encoded and epigenetically regulated, while bacterial small RNAs may mimic miRNA functions through host-pathogen co-evolution.

Extracellular vesicles secreted by *P.acnes* have been demonstrated to cause acne-like dermatitis *in vitro* skin model. These vesicles regulate epidermal differentiation by promoting keratinocyte proliferation, reducing epidermal keratin 10 and desmocollin 1 levels, and inducing the inflammatory factors interleukin 8 (IL-8) and granulocyte-macrophage colony-stimulating factor (GM-CSF) (9). Keratinocytes absorb extracellular vesicles, activating transient receptor potential vanilloid 1(TRPV1) and increasing inflammatory factors expression, including IL-6, IL-8, and TNF-α through ERK1/2 MARK and NF-κB pathways (10). The researchers have discovered that miRNAs such as hsa-miR-155, hsa-miR-223, hsa-miR-21, and hsa-miR-146a, present in tissues, cells, and blood circulation, are associated with acne vulgaris (11). Expression levels of hsa-miR-21 and hsa-miR-223 were elevated in acne scars, while hsa-miR-155 was rapidly up-regulated during the initial stages of acne inflammation. *P.acnes* can promote miR-146 expression in keratinocytes, while TLR1/2–4 activation in sebocytes may induce miR-146 expression. *P.acnes* and *Staphylococcus epidermidis* co-colonize acne lesions (12), triggering inflammatory factors by TLR2 activation. Lipoteichoic acid (LTA), released by *Staphylococcus epidermidis*, activates mir-143 in TLR2-induced keratinocytes, thereby inhibiting TLR2 protein expression. MiRNA expression profiles in the peripheral blood and skin lesions of patients with acne suggest that certain miRNAs could serve as biomarkers for acne and acne-related depressed scars. Three miRNAs (miR-223, miR-21, and miR-150) were significantly up-regulated in acne lesions, particularly in non-lesional skin, which has a higher susceptibility for scarring. Additionally, elevated miR-21 and miR-150 expression levels were observed in the peripheral blood of acne patients (13). The adipogenesis of sebaceous gland cells treated with hsa-miR-338-3p was shown to decrease, alongside reduced phosphorylation of Akt, a key component of the PIK3-Akt pathway, suggesting that miR-338-3p may regulate the PIK3-Akt pathway to reduce sebogenesis (14). Research indicates that *P.acnes* treatment down-regulates hsa-miR-196a in keratinocytes while up-regulating TLR2. MiR-196a mimics reduce TLR2 expression and activate NF-κB in keratinocytes (15). Evidence from previous studies indicates a correlation between elevated miRNA levels and the inflammatory response associated with acne (**Supplementary Figure 1**).

Recent data suggest that extracellular vesicles derived from *P.acnes* may contribute to acne development. The functional status of exosomes from acne skin lesions and peripheral blood remains unclear. MiRNAs from blood circulation, tissues, and cells contribute to the inflammatory immune response in acne as pro-inflammatory molecules. Whether these miRNA molecules are encapsulated in exosomes and transported to the circulation and related skin tissues remains unknown. No studies have yet examined the expression profile of miRNAs in exosomes. Further research is needed to deepen understanding of miRNA expression in acne.

## Materials and methods

### Patients and controls

15 patients with severe acne and 15 age- and gender-matched healthy individuals included this study. The inclusion criteria of experimental group were as follows: (1) acne clinically diagnosed as severe according to Pillsbury (Grade IV) (16) and global acne grading system (GAGS) (17); (2) age 14–40 years, without concomitant inflammatory or autoimmune dermatological conditions, systemic disorders, or malignant neoplasms; (3) no oral isotretinoin therapy in the last three months and no oral antibiotics, glucocorticoids, or contraceptives in the past one month. The exclusion criteria included pregnancy, lactation or any systemic comorbidities. The inclusion criteria of control group were: (1) no prior history of acne vulgaris, and (2) age 14–40 years, without dermatological conditions, systemic disorders, or malignant neoplasms. Exclusion criteria were as above.

### Isolation of exosomes from serum

Exosome were isolated from serum using Ribo™ exosome Isolation reagent (Ribobio, China). Peripheral blood (10 ml) was centrifuged at 1000-2000×g for 10 min, followed by 3000×g 4°C for 10 min and 10000×g 4°C for 30 min to remove impurities and cell debris. After the preliminary separation, the serum was centrifuged at 2000×g for 20 min, mixed with 1/3 volume of Ribo™ exosome Isolation reagent, and centrifuged at 15000×g 4°C for 2 min. The obtained precipitated part contained the exosomes.

### Exosome identification

Exosome markers CD9, CD63, CD81 and TSG101 were verified by Western blotting. Exosome size and concentration were assessed using nanoparticle tracking analysis (NTA) with ZetaView (Particle Metrix, Germany), while exosome shape was evaluated by Transmission Electron Microscopy (TEM, HITACHI, Japan).

### RNA extraction and miRNA sequencing

Total RNA was extracted from serum exosomes using the HiPure Liquid miRNA Kit/HiPure Serum/Plasma miRNA (Magen, China). RNA concentration was analyzed by qubit 2.0, and the distribution of RNA fragments was detected by Agilent 2200 TapeStation (Agilent Technologies). After constructing miRNA libraries, cDNA libraries were produced with the NEBNext Multiplex Small RNA Library Prep Set for Illumina (NEB, USA) and sequenced on the Illumina HiSeq 2500 platform.

### Analysis of differentially expressed miRNAs

DESeq2 R package was used to identify differentially expressed miRNAs in serum exosomes between experimental group and control group. The screening criteria including | log2 (FoldChange) | > 1 and significant level (*P* < 0.05). The DESeq2 R package was used to generate volcano map, heat map, and differential scatter plots.

### Target genes prediction of miRNAs

Target genes of miRNAs were predicted using Target Scan 8.0 (https://www.targetscan.org/vert_80/), miRDB6.0 (https://mirdb.org/), miRTarBase9.0 (https://mirtarbase.cuhk.edu.cn/), and miRWalk3.0 (http://mirwalk.umm.uni-heidelberg.de/). Target gene selection criteria were as follows: (1) genes consistent with the miRTarBase database; (2) genes that concur with miRWalk, TargetScan, miRDB database; (3) meeting either criterion (1) or (2).

### GO, and KEGG pathway enrichment analyses

All predicted target genes were uploaded to the DAVID database (https://david.ncifcrf.gov/), and Gene Ontology (GO) analysis and Kyoto Encyclopedia of Genes and Genomes (KEGG) studies were conducted using the Functional Annotation Tool, screening for enriched entries with P<0.05. GO and KEGG enrichment analyses were displayed and plotted by the RiboBio Galaxy platform (Ribobio, China).

### Screening of miRNA hub genes

The target gene entries were submitted into the String database (https://cn.string-db.org/) with the “minimum required interaction score” configured to 0.9 to generate the PPI interaction network which was loaded into cytoscape3.10.0. Hub genes were identified by intersecting results from the cytoHubba and MCODE plug-ins in Cytoscape 3.10.0. Mapping miRNA-target gene interactions using cytoscape 3.10.0.

### Real-time quantitative PCR assessment of exosome miRNA

The selected target miRNAs were quantified in each sample by RT-qPCR using the Bulge-Loop™ miRNA qRT-PCR kit (Ribobio, China), employing cel-miR-39-3p as an external reference. Total RNA (3μl) was reverse-transcribed in a 10μl reaction volume, and the RT-qPCR assay was performed using a dye-based detection system.

## Result

### Identification and characterization of exosomes

**Figure 1:**
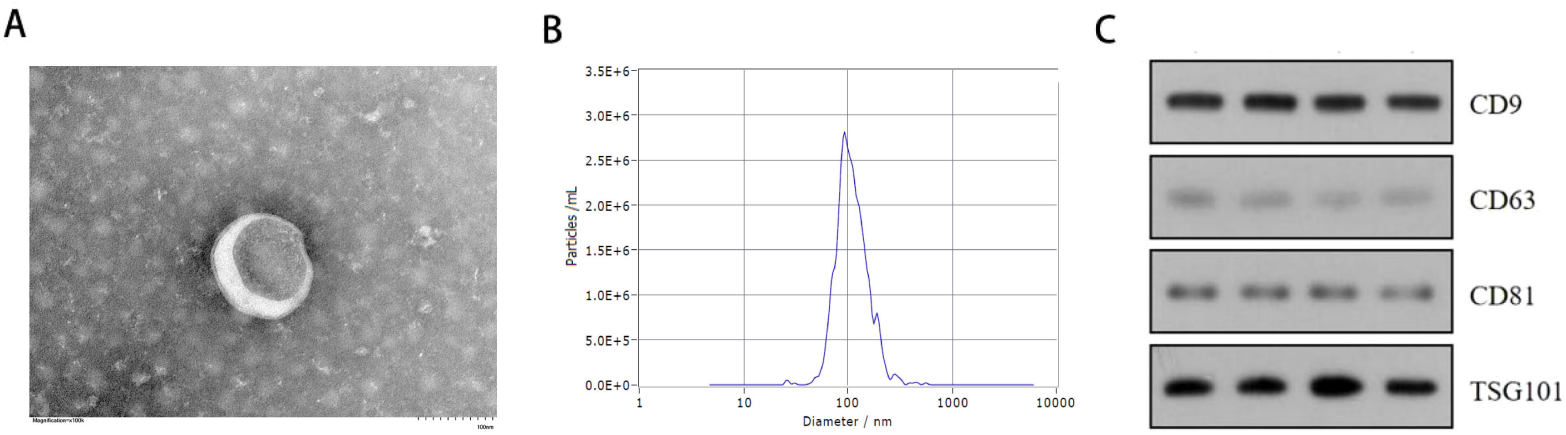
Exosome identification results. **(A)** Transmission electron microscope (TEM) analysis of exosomes (Scale bars, 100 nm). **(B)** Nanoparticle tracking analysis (NTA) showing the size distribution of exosomes purified from the serum. **(C)** Western blot analysis of exosome specific surface markers (CD63,CD9,CD81,TSG101) in serum exosome of 2 patients with severe acne and 2 healthy individuals.

Serum-derived vesicles were initially examined by transmission electron microscopy (TEM), revealing a typical saucer-shaped morphology of exosomes (**Figure 1A**). The median particle size of the vesicles detected by the nanoparticle tracking analysis (NTA) was 103.8nm, with a primary peak of particle size at 93.6 nm, consistent with typical exosome dimensions (**Figure 1B**). Western blot confirmed the presence of exosome markers CD63, CD81, CD9, and TSG101 in all serum samples examined (**Figure 1C**).

### Serum exosomal differentially expressed miRNAs

**Figure 2:**
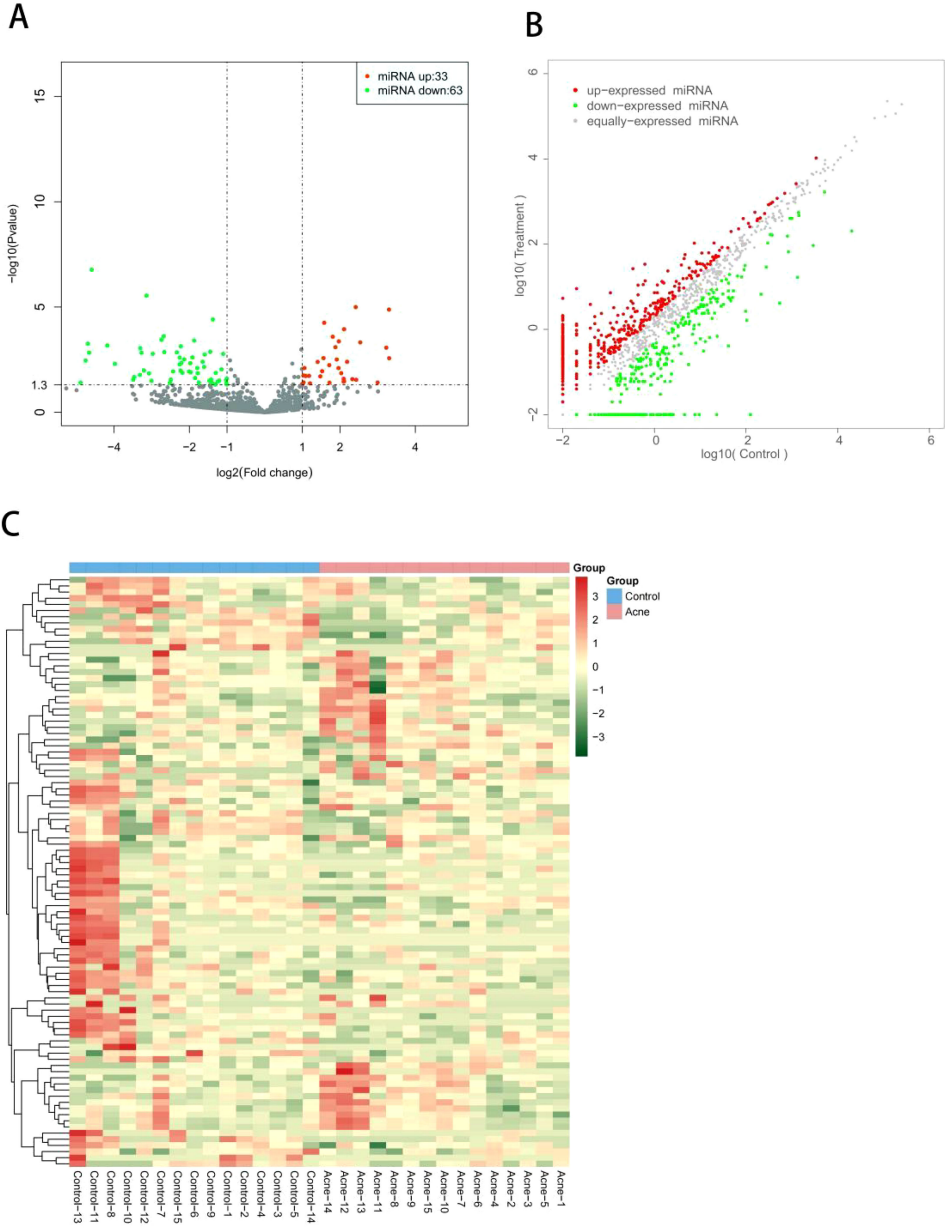
**(A)** Volcano plot of differentially expressed miRNAs between patients with severe acne vulgaris and healthy controls. All miRNA levels are displayed, with differently expressed miRNAs indicated in red (up-regulated) or green (down-regulated). **(B)** Scatter plot of differentially expressed miRNAs between patients with severe acne vulgaris and healthy controls. **(C)** Heat map of differentially expressed miRNAs between patients with severe acne vulgaris and healthy controls.

Based on the second-generation sequencing, significantly different expression profile of miRNAs in the serum exosomes of the two groups are shown. Compared to the control group, 96 differentially expressed miRNAs were identified in acne group, with 33 up-regulated and 63 down-regulated, including hsa-miR-6529-5p, hsa-miR-105-5p, hsa-miR-9-5p, hsa-miR-124-3p, hsa-miR-1-3p, hsa-miR-219a-2-3p, hsa-miR-383-5p, hsa-miR-889-3p, hsa-miR-9-3p, and hsa-miR-3168. Besides, top differential miRNAs between acne patients and healthy controls were stated in **Supplementary Table 1**. Two thirds of all differentially expressed miRNAs were down-regulated, as were the top 15 miRNAs. Of these 96 miRNAs, most of them were newly identified in severe acne while only miR-21-3p have been previously reported in human acne. The overall distribution of these miRNAs is shown in volcano plots (**Figure 2A**) and scatter plots(**Figure 2B**), while a heat map illustrates the clustering relationship of miRNAs expression among samples (**Figure 2C**).

### Prediction of target genes for differently expressed miRNAs

Target genes of the 96 differentially expressed miRNAs were predicted using TargetScan, miRDB, miRTarBase, and miRWalk databases. After databases interrogation, more than 10,000 genes were found to be targeted by these 96 miRNAs. The number of target genes for the top 10 differentially expressed miRNAs were shown in **Table 1**. There were 217 predicted target genes for miR-6529-5p, 470 predicted target genes for miR-105-5p, 607 predicted target genes for miR-9-5p, 1467 predicted target genes for miR-124-3p and 958 predicted target genes for miR-1-3p. And miR-124-3p has the most target genes. After sorting and counting, 10,569 target genes were identified. Some target genes were likely involved in acne development, including AR, IGF1/IGF1R, mTOR, GATA6, Wnt, IL6, FOXO1, PIK3, MYC.

**Table 1:** The number of target genes for the top 10 differentially expressed miRNAs.

miRNA	log2(Fold_change)	Count
hsa-miR-6529-5p	-8.066743234	217
hsa-miR-105-5p	-6.586143592	470
hsa-miR-9-5p	-6.254990415	607
hsa-miR-124-3p	-6.223095804	1467
hsa-miR-1-3p	-5.916477522	958
hsa-miR-219a-2-3p	-4.89024682	92
hsa-miR-383-5p	-4.756763034	96
hsa-miR-889-3p	-4.696725191	128
hsa-miR-9-3p	-4.660888558	134
hsa-miR-3168	-4.594956563	125
……		

### GO, and KEGG pathway enrichment analyses

**Figure 3:**
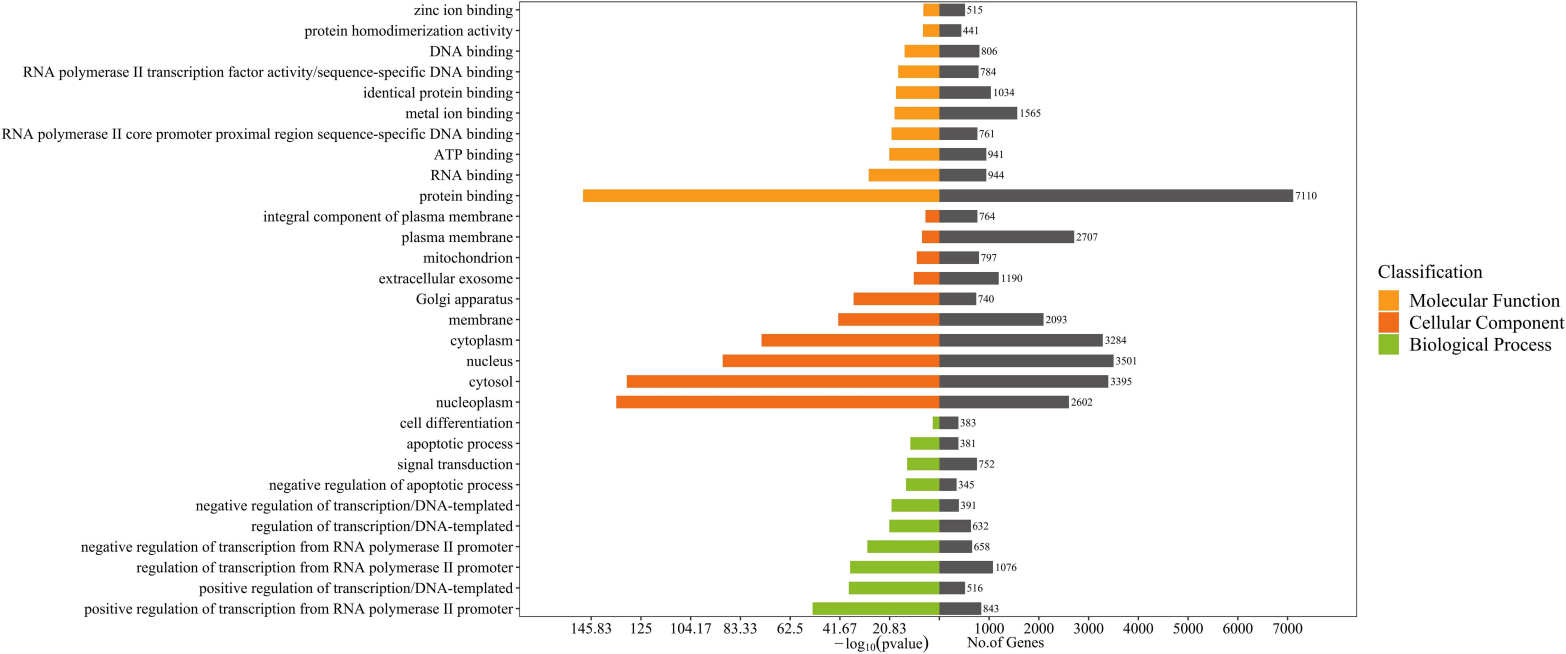
GO enrichment analysis for target genes of 96 differentially expressed miRNAs detected in acne patient serum exosomes (*P*<0.05). Bars are ranked by -log_10_ (pvalue).

Gene Ontology (GO) annotation for the target genes identified a total of 1303 biological process annotations, 252 cellular component annotations, and 284 molecular function annotations (*P*<0.05). Biological process (BP) were mainly involved in transcriptional regulation and signal transduction; cellular component (CC) included the nucleus, cytoplasm, membrane and nucleoplasm; molecular function (MF) were mainly related to protein binding, RNA binding,and ATP binding (**Figure 3**).

**Figure 4:**
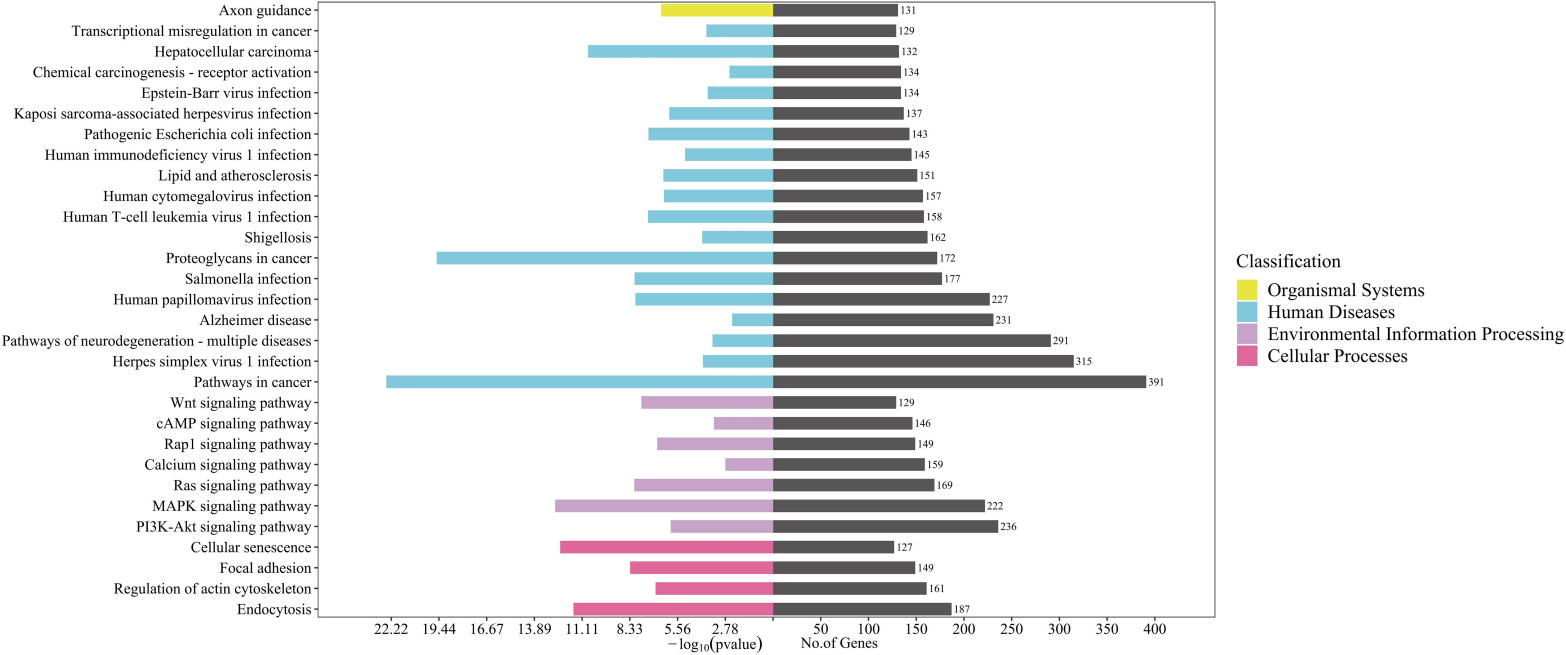
KEGG pathway enrichment analysis for target genes of 96 differentially expressed miRNAs detected in acne patient serum exosomes (*P*<0.05). Bars are ranked by enrichment terms.

KEGG enrichment analysis showed that target genes were significantly enriched in 160 pathways, including pathways in cancer, herpes simplex virus 1 infection, pathways of neurodegeneration-multiple diseases, phosphatidylinositol-3,4,5-trisphosphate-dependent protein kinase B (PI3K-Akt) signaling pathway, alzheimer disease, human papillomavirus infection, and mitogen-activated protein kinase (MAPK) signaling pathway etc (*P* < 0.05) (**Figure 4**). KEGG secondary classification findings suggested that the target genes were primarily involved in signal transduction pathways (**Supplementary Figure 2**). We further screened 14 signaling pathways related to the pathogenesis of acne, including PI3K-Akt signaling pathway and MAPK signaling pathway (**Table 2**).

**Table 2:** miRNA target genes and the KEGG-enriched pathway linked with acne vulgaris.

Rank	KEGG number	Term	Count	%	P-Value
4	hsa04151	PI3K-Akt signaling pathway	236	2.36	1.09E-06
7	hsa04010	MAPK signaling pathway	222	2.215568862	2.07E-13
28	hsa04310	Wnt signaling pathway	129	1.28742515	2.17E-08
32	hsa04150	mTOR signaling pathway	123	1.22754491	6.03E-11
36	hsa04062	Chemokine signaling pathway	121	1.20758483	0.005453539
45	hsa04910	Insulin signaling pathway	107	1.067864271	3.33E-09
50	hsa04068	FoxO signaling pathway	103	1.027944112	3.55E-09
62	hsa04152	AMPK signaling pathway	93	0.928143713	1.61E-07
73	hsa04350	TGF-beta signaling pathway	86	0.858283433	2.93E-08
80	hsa04931	Insulin resistance	77	0.768463074	1.91E-04
85	hsa04659	Th17 cell differentiation	75	0.748502994	8.22E-04
89	hsa04064	NF-κB signaling pathway	73	0.728542914	6.05E-04
106	hsa04620	Toll-like receptor signaling pathway	68	0.678642715	0.039477706
112	hsa04115	p53 signaling pathway	63	0.628742515	2.83E-08

### Protein–protein interaction network

**Figure 5:**
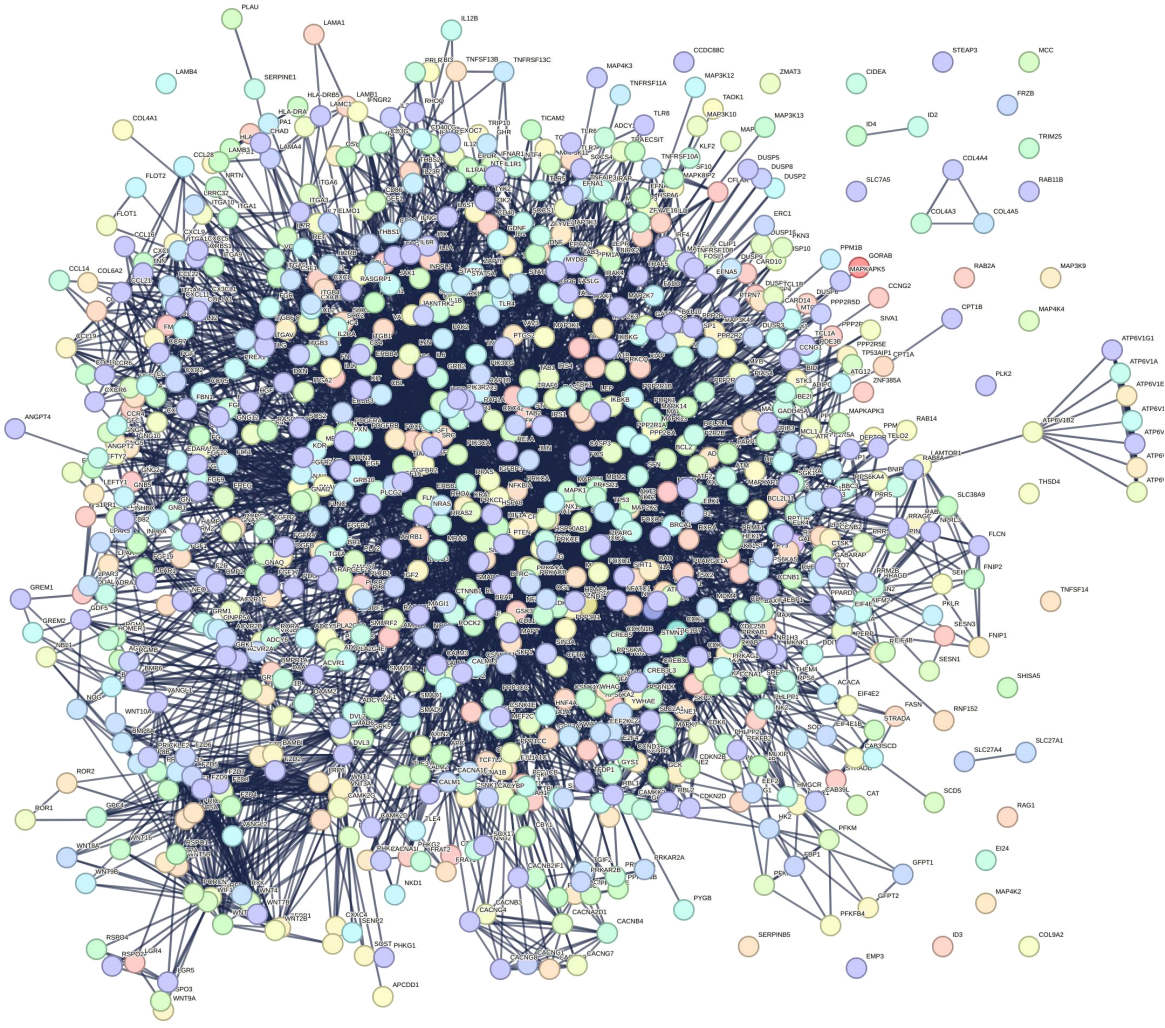
Protein-protein interaction (PPI) network. The network has 894 nodes and 6,332 edges, with an average node degree of 14.2.

A total of 1576 target genes from the aforementioned 14 KEGG enrichment pathways were submitted to the String database to generate the PPI interaction network seen in the following graph (**Figure 5**). The network comprised 894 nodes and 6332 edges, with an average node degree of 14.2.

### Screening of miRNA hub genes

**Figure 6:**
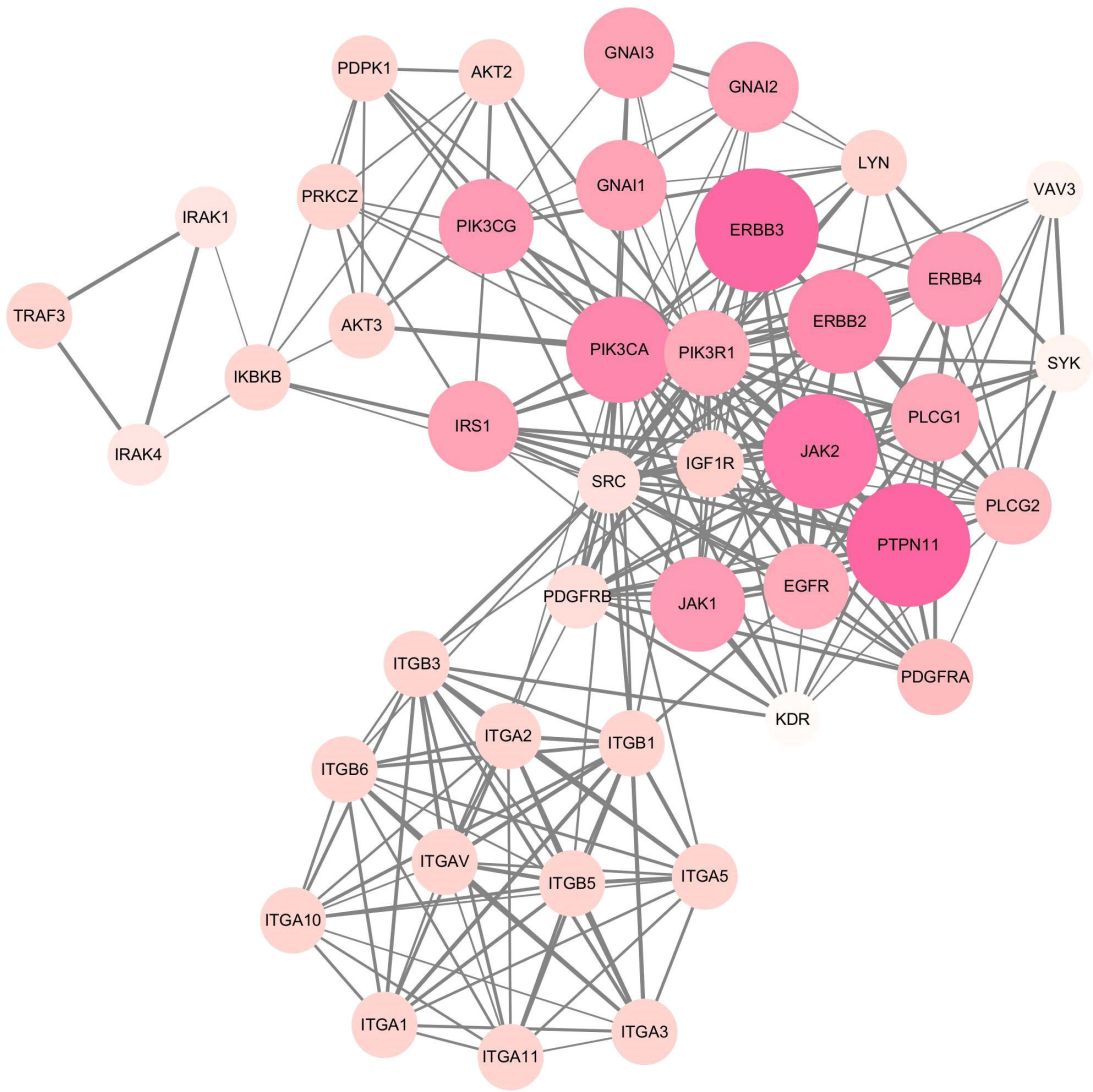
Hub module 1 composed of 43 nodes connected by 248 edges, screening by MCODE plug-in.

**Figure 7:**
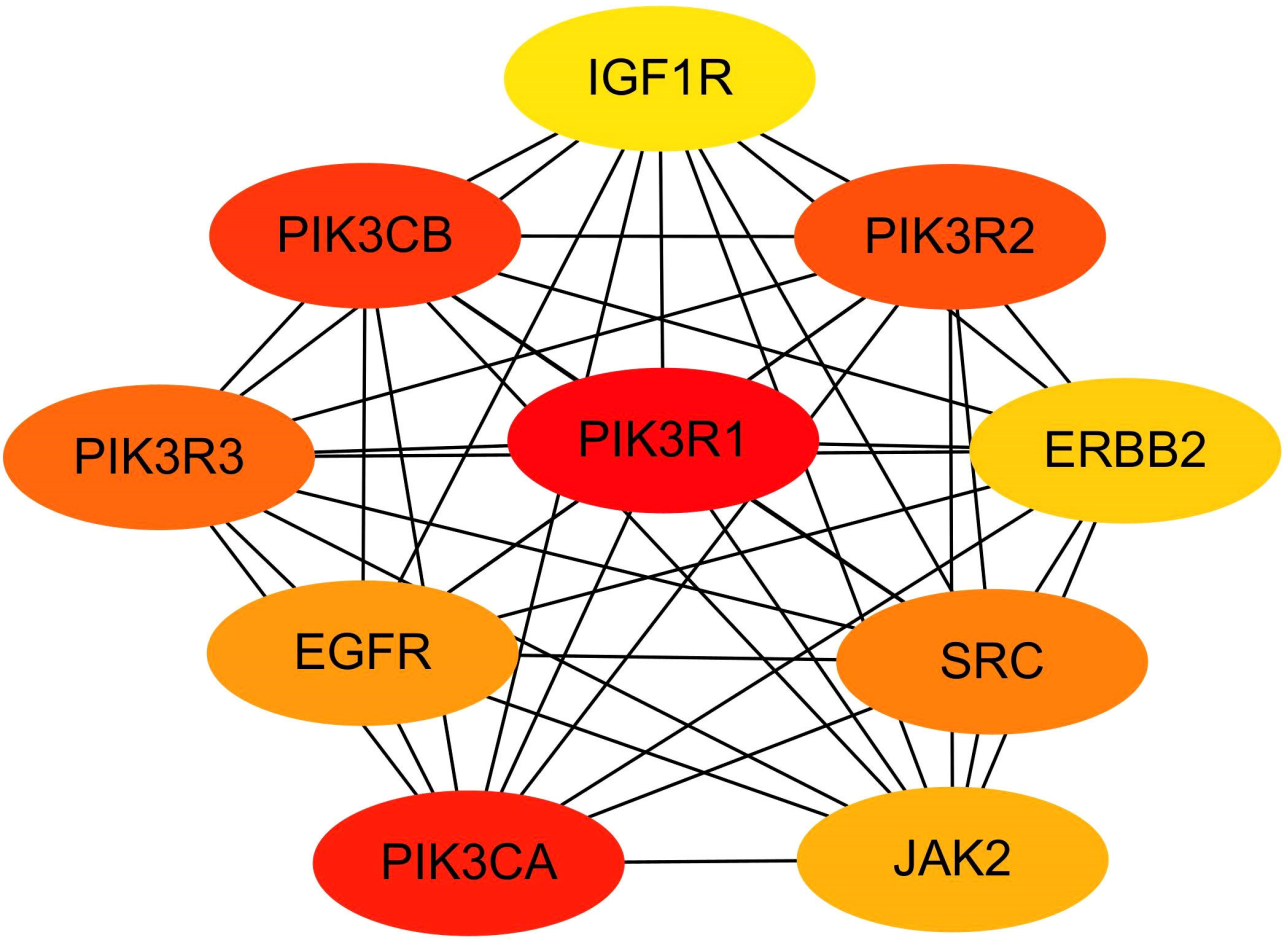
Hub module 2 consisted of top-scroing 10 nodes ranked in Cytohubba plug-in.

MCODE plug-in screening in Cytoscape3.10.0 showed that the sub-network with the highest network score (11.810 points) contained 43 nodes. Ultimately, 43 hub genes were identified, including PTPN11, ERBB2, ERBB3, JAK2, PIK3CA, and PIK3R1 (**Figure 6**). Utilizing the Cytohubba plug-in, we identified top-scoring ten nodes (target genes), including PIK3R1, PIK3CA, ERBB2, EGFR, and IGF1R (**Figure 7**).

### miRNA-target gene interaction

**Figure 8:**
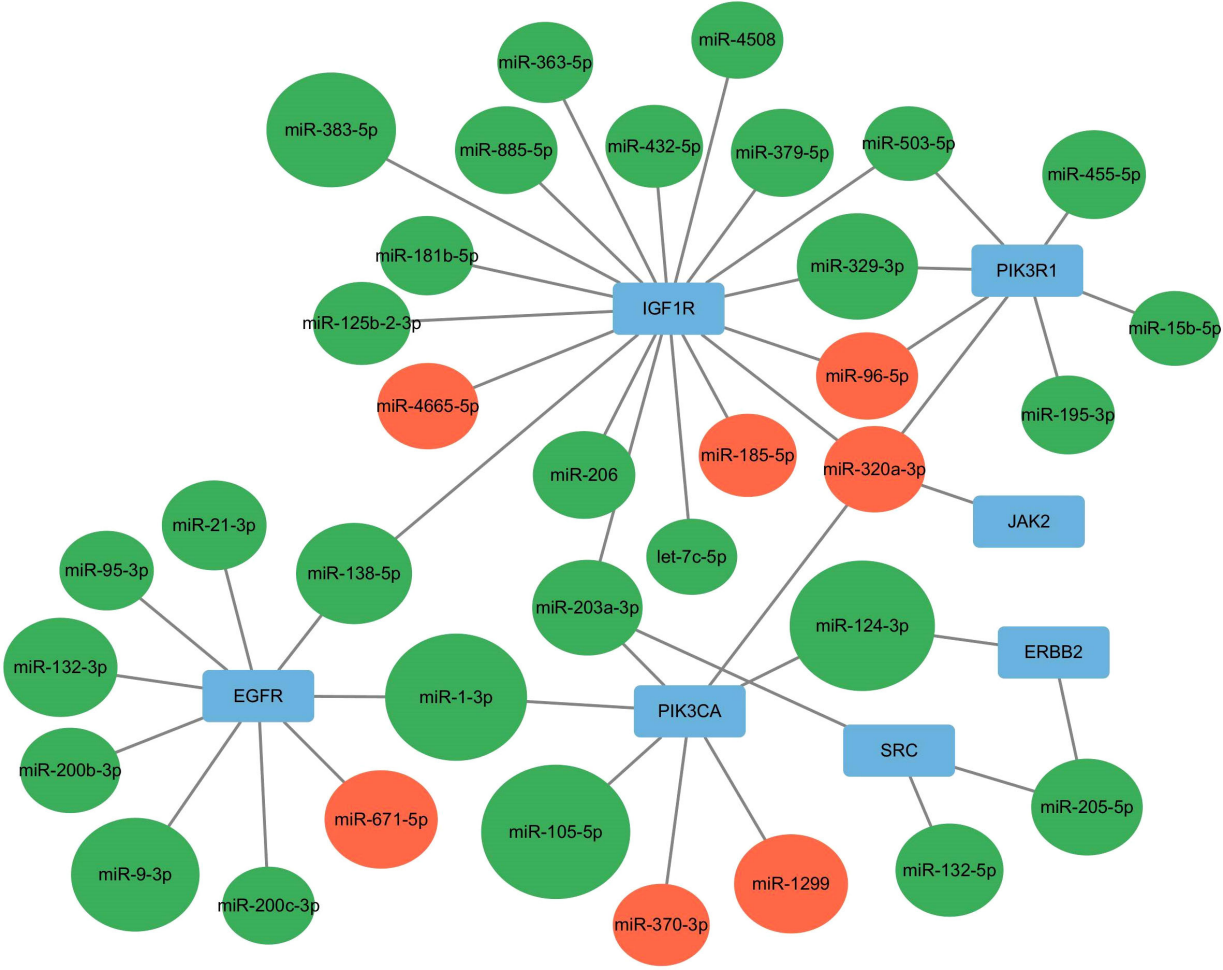
miRNA-Hub genes interaction network. Red indicates up-regulated miRNA, whereas green signifies down-regulated miRNA.

Intersecting the hub genes identified by the two plug-ins revealed seven hub genes: PIK3R1, PIK3CA, SRC, EGFR, JAK2, ERBB2, and IGF1R. These hub genes corresponded to 35 target-regulated miRNAs, with miRNA-target gene interaction networks visualized using Cytoscape 3.10.0 (**Figure 8**).

### RT-qPCR assessment of exosome miRNA

**Figure 9:**
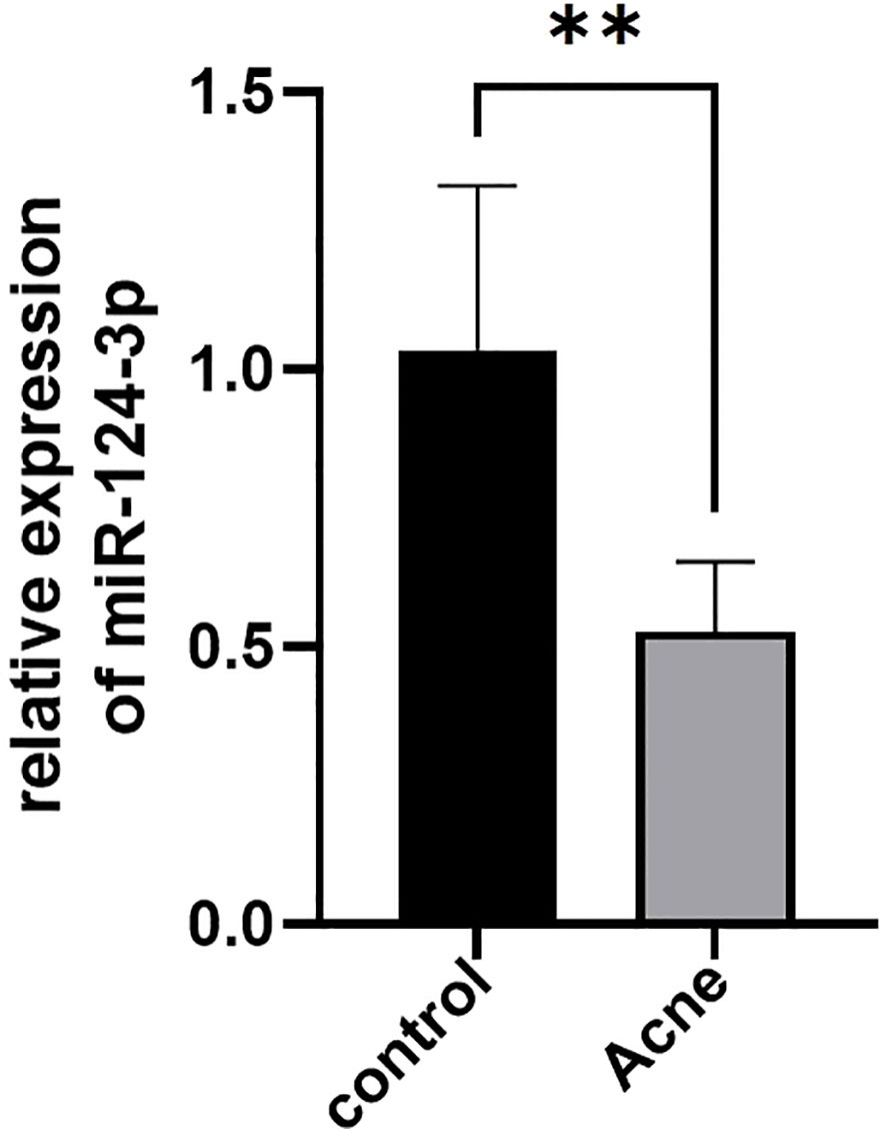
Differential expression of serum exosomal miR-124-3p in patients with severe acne vulgaris (n=5) and healthy controls (n=5) tested by RT-qPCR. Results are expressed as mean ± SD. **P<0.01, unpaired t-test.

The top 5 most differentially expressed miRNAs (miR-105-5p, miR-124-3p, miR-1-3p, miR-383-5p, miR-9-3p) among 35 differentially expressed miRNAs were selected for RT-qPCR validation (**Table 3**). MiR-124-3p expression was significantly decreased in the severe acne group compared to the healthy controls (*P*<0.05, **Figure 9**), consistent with the results in small RNA sequencing. The expression levels of the remaining four miRNAs were found to be too low in both groups, which is not typical.

**Table 3:** Hub genes corresponds to the targeted top five differentially expressed miRNAs.

Rank	miRNA	log2(Fold_change)	Genes
1	miR-105-5p	-6.586143592	PIK3CA
2	miR-124-3p	-6.223095804	PIK3CA, ERBB2
3	miR-1-3p	-5.916477522	PIK3CA, EGFR
4	miR-383-5p	-4.756763034	IGF1R
5	miR-9-3p	-4.660888558	EGFR

## Discussion

Acne, affecting approximately 85% of teenagers (18), is driven by inflammatory processes in the sebaceous gland units of hair follicles (19). Emerging therapeutic strategies are targeting dysregulated signaling networks and key inflammatory mediators (20). Exosomes mediate intercellular communication by transferring miRNAs (21). Exosomal miRNAs are increasingly recognized for their diagnostic potential across a wide range of diseases (22). Advances in second-generation sequencing and exosome isolation technologies have made it possible to reveal distinct exosomal miRNA expression profiles for various disease models (23).

In this study, we demonstrated dysregulation of a number of miRNAs in acne patients. A reduction in ‘anti-inflammatory miRNAs’ is more likely associated with the onset of severe acne, as two-thirds of the miRNAs were downregulated, with 25 of the top 30 miRNAs exhibited downregulation in severe acne cases. Through bioinformatics analysis of target gene prediction and core gene screening, we identified seven hub genes: PIK3R1, PIK3CA, SRC, EGFR, JAK2, ERBB2, and IGF1R. These seven hub genes play a central role and form the core of the network of miRNA-target gene interactions in severe acne. RT-qPCR validation confirmed that patients with severe acne had significantly lower levels of miR-124-3p expression.

MiR-124-3p, a highly conserved microRNA (24), has been implicated in the pathogenesis of various autoimmune diseases, tumors, and neurological disorders (25). Evidence indicates that miR-124 is involved in neural stem cells formation and is enriched in neuron-derived exosomes, where it suppresses the activation of M1-type microglia and A1-type astrocytes, mitigating spinal cord injury (26). In cancer, miR-124-3p promotes breast cancer progression by targeting the Axin1 protein, facilitating cancer cell proliferation (27). Downregulation of miR-124-3p in gastric cancer correlates with advanced clinical stage, lymph node metastasis, and worse prognosis (28).

The miRNA database demonstrates that miR-124 is mostly present in human neurological tissues, including the arachnoid, dura mater, brain tissue, and spinal cord. However, studies increasingly explore the role of miR-124 in dermatology. In vitiligo, miR-124-3p was found to distinguish patients from healthy individuals with an area under the curve (AUC) of 0.835 (29). MiR-124 also exhibits reduced expression in cancerous tissues, where it inhibits melanoma growth, metastasis, and promotes apoptosis by targeting RACK1 (30). In individuals with toxic epidermal necrolysis (TEN), miR-124 expression significantly increases and correlates with the extent of epidermal exfoliation and SCORTEN scores. Moreover, a significant correlation was seen between miR-124 expression and the extent of epidermal exfoliation, as well as the SCORTEN score in TEN. Serum miR-124 may function as a potential marker of disease activity in severe drug eruptions (31). Additionally, miR-124 influences atopic dermatitis development by regulating inflammatory protein synthesis via the NF-κB signaling pathway in keratinocytes (32). While miR-124 expression is reduced in malignant tumors and inflammatory dermatoses, it is elevated in disorders of melanin metabolism and dermatitis medicamentosa. Although miR-124 has been studied in various dermatological disorders, no research has yet investigated its exosomal expression in dermatological diseases or acne vulgaris.

Our study revealed a down-regulation of miR-124-3p in severe acne, suggesting that miR-124-3p may act as an anti-inflammatory agent. The KEGG secondary classification findings showed that the target genes are predominantly enriched in signal transduction pathways, particularly in the PI3K-Akt pathways. Recent studies show that androgens, insulin, and IGF-1 promote sebum secretion and acne development by activating the PI3K-Akt pathway (33) This pathway suppresses FOXO1 (34)upregulates mTORC1/SREBP-1 signaling, and triggers sebocyte proliferation, inflammation, and adipogenesis, ultimately contributing to acne pathogenesis (35). Aberrant PI3K-Akt plays a crucial role in the etiology of acne, and can lead to inflammation. Exosomal miR-124-3p has been found to prevent the progression of non-small cell lung cancer by blocking the PI3K-Akt signaling pathway (36). Researchers found that acute ischemic stroke reduced serum exosomal miR-124-3p expression, which in turn facilitated the phosphorylation of PI3K-Akt (37). These findings suggest that miR-124 participates in inflammatory processes linked to the PI3K-Akt pathway. Of note, PIK3CA seems to be an important target miR-124-3p. In hepatocellular carcinoma (HCC), miR-124-3p directly targets PIK3CA to suppress tumor proliferation by attenuating PI3K-Akt signaling (38). Additionally, treatment with IGF-1 increased the gene expression of PIK3CA and also sebum production in cultured sebocyte (39). It is currently known that PIK3CA-driven PI3K/Akt/mTOR signaling is a master regulator of lipid synthesis and gland homeostasis. Does this miR-124-3p/PIK3CA regulatory axis may extend to skin pathologies?

According to KEGG data, most of the seven hub genes are within the PI3K-Akt pathway and are functionally related to oncogenes, primarily regulating cell proliferation, differentiation, and other physiological processes. The myelocytomatosis oncogene (c-MYC) is one of the most extensively studied oncogenes and has been shown to act as a significant transcription factor in sebaceous cell development (40). c-MYC upregulation markedly suppresses p53 transcriptional activity, promoting survival and differentiation of sebum cells. Moreover, c-MYC directly stimulates androgen receptor (AR) expression (41). Although there are no reports linking these ‘oncogenes’ directly to acne vulgaris, they are part of the PI3K-Akt pathway, and dysregulated activation of this pathway has been associated with acne pathogenesis. It is thus relevant to explore whether these hub genes, like c-MYC, emerges as a pivotal regulatory hub not only in systemic cancers but also in cutaneous biology to influence sebaceous gland cell or keratinocyte differentiation and proliferation and contribute to acne.

In general, our study is the first to reveal the serum exosome miRNA profiles of severe acne compared to healthy controls, demonstrating a significant down-regulation of miR-124-3p in severe acne. Our findings provide a new understanding of acne pathogenesis. MiR-124-3p holds promise for clinical translation including diagnostic applications and therapeutic targeting. Though several studies in the literature substantiate the involvement of miR-124-3p in the PI3K-Akt pathway, further experimental validation is necessary to ascertain the involvement of miR-124-3p in the PI3K-Akt pathway and its regulatory effects on target genes within this pathway. In addition, assessing exosomal miRNA levels in acne tissue samples may improve our comprehension of the role of miRNAs in acne pathogenesis. Therefore, further specific study are needed, and also multi-center studies with larger cohorts are needed to validate these biomarkers.

## Data Availability

The original contributions presented in the study are included in the article/**Supplementary Material**, further inquiries can be directed to the corresponding authors.

## Supplementary material

Supplementary Figure 1 Molecular mechanisms of P.acnes-derived extracellular vesicles and miRNAs in regulating keratinocyte&sebocyte-driven inflammatory responses in acne. Abbreviations: P.acnes, propionibacterium acnes; IL-6, interleukin 6; IL-8, interleukin 8; TNF-α, tumor necrosis factor-alpha; GM-CSF, granulocyte-macrophage colony-stimulating factor; ERK, extracellular regulated protein kinase; MARK, mitogen-activated protein kinase; TLR2, Toll-like receptor 2; K10, Keratin 10; DSC1, desmocollin 1; PIK3-Akt, phosphatidylinositol 3-kinase–protein kinase B; TRPV1, transient receptor potential vanilloid type-1; NF-κB, nuclear factor-κB.

Supplementary Figure 2 Enrichment of target genes for miRNA KEGG pathway secondary classification diagram.

Supplementary Table 1 Top 30 differential miRNAs between the severe acne patients and the health controls.
