# Supplementary figures and images for: Serum exosomal microRNA profiling reveals a down-regulation of hsa-miR-124-3p in patients with severe acne
Source: Front Immunol. 2025 Jun 23;16:1554811. doi: 10.3389/fimmu.2025.1554811 (PMC12230035; doi:10.3389/fimmu.2025.1554811)

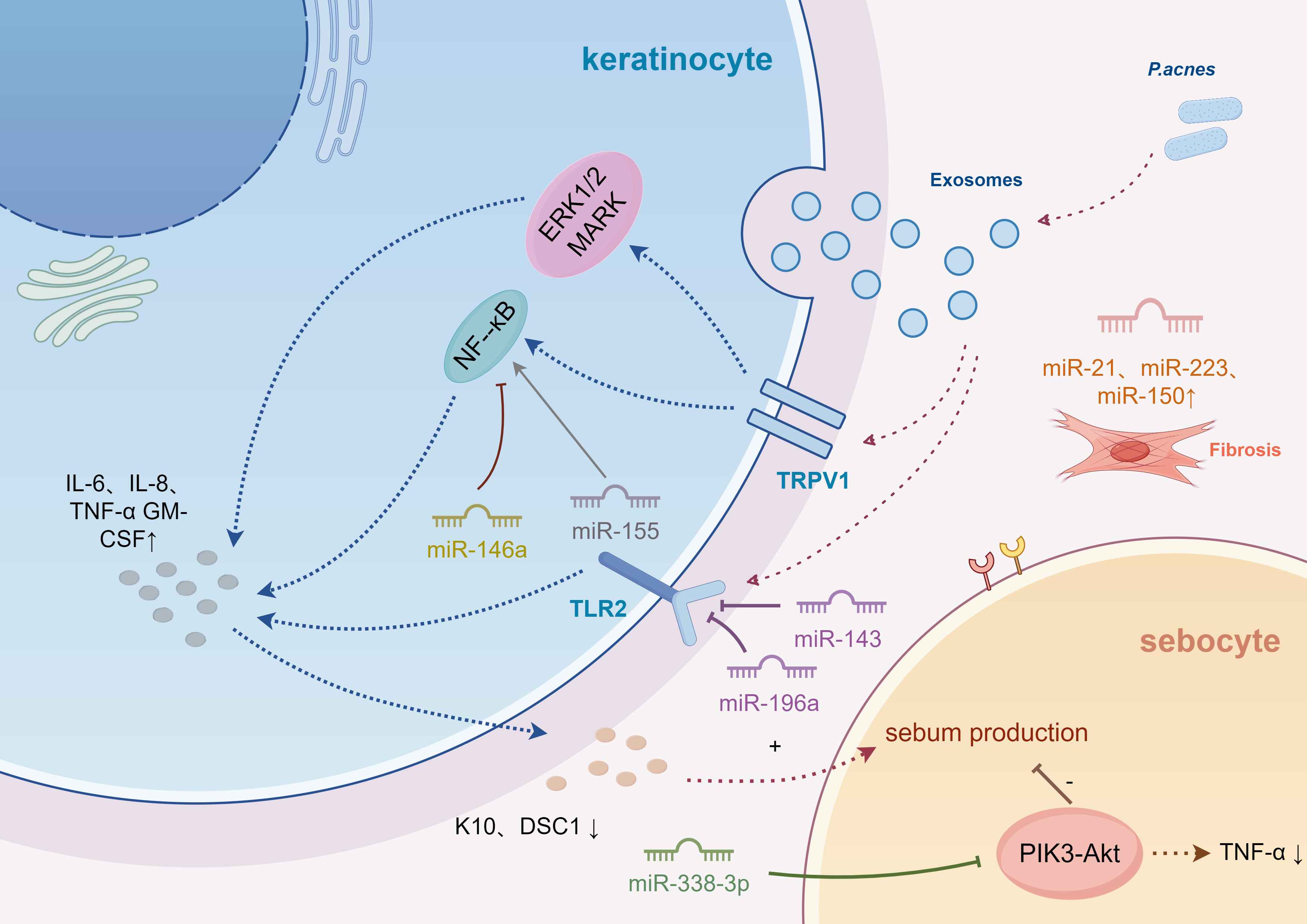

Supplement: Supplementary Figure 1 — Molecular mechanisms of P.acnes-derived extracellular vesicles and miRNAs in regulating keratinocyte&sebocyte-driven inflammatory responses in acne. Abbreviations: P.acnes, propionibacterium acnes; IL-6, interleukin 6; IL-8, interleukin 8; TNF-α, tumor necrosis factor-alpha; GM-CSF, granulocyte-macrophage colony-stimulating factor; ERK, extracellular regulated protein kinase; MARK, mitogen-activated protein kinase; TLR2, Toll-like receptor 2; K10, Keratin 10; DSC1, desmocollin 1; PIK3-Akt, phosphatidylinositol 3-kinase–protein kinase B; TRPV1, transient receptor potential vanilloid type-1; NF-κB, nuclear factor-κB. [file Image1.tiff]

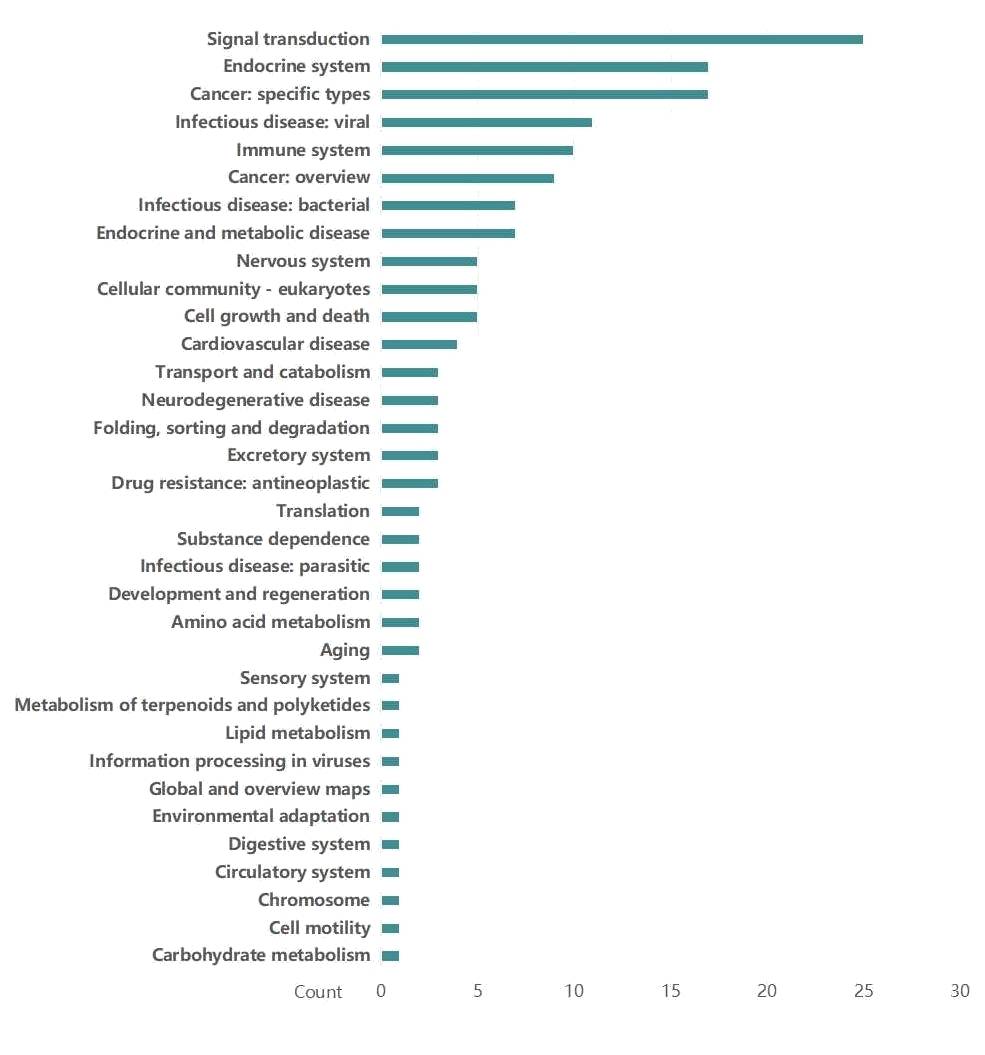

Supplement: Supplementary Figure 2 — Enrichment of target genes for miRNA KEGG pathway secondary classification diagram. [file Image2.tiff]
