# Supplementary material for: Serum exosomal microRNA profiling reveals a down-regulation of hsa-miR-124-3p in patients with severe acne
Source: Front Immunol. 2025 Jun 23;16:1554811. doi: 10.3389/fimmu.2025.1554811 (PMC12230035; doi:10.3389/fimmu.2025.1554811)
Supplement: Supplementary Table 1 — Top 30 differential miRNAs between the severe acne patients and the health controls. [file Table1.docx]

| miRNA | log2(Fold change) | p-value | Regulation |
| --- | --- | --- | --- |
| hsa-miR-6529-5p | -8.066743234 | 0.005932457 | down |
| hsa-miR-105-5p | -6.586143592 | 0.024848885 | down |
| hsa-miR-9-5p | -6.254990415 | 0.000372309 | down |
| hsa-miR-124-3p | -6.223095804 | 0.004534523 | down |
| hsa-miR-1-3p | -5.916477522 | 1.24E-08 | down |
| hsa-miR-219a-2-3p | -4.89024682 | 0.038836788 | down |
| hsa-miR-383-5p | -4.756763034 | 0.003477185 | down |
| hsa-miR-889-3p | -4.696725191 | 0.000550527 | down |
| hsa-miR-9-3p | -4.660888558 | 0.001464381 | down |
| hsa-miR-3168 | -4.594956563 | 1.68E-07 | down |
| hsa-miR-136-3p | -4.179817462 | 0.000671591 | down |
| hsa-miR-329-3p | -3.977539272 | 0.004978285 | down |
| hsa-miR-138-5p | -3.489845591 | 0.028415458 | down |
| hsa-miR-6511b-3p | -3.466942929 | 0.021100664 | down |
| hsa-miR-6513-3p | -3.313174393 | 0.016889678 | down |
| hsa-miR-6807-5p | 3.305352033 | 0.002704274 | up |
| hsa-miR-132-3p | -3.304546029 | 0.000907477 | down |
| hsa-miR-1299 | 3.298648166 | 1.32E-05 | up |
| hsa-miR-365a-5p | -3.271173791 | 0.001491574 | down |
| hsa-miR-671-5p | 3.228395985 | 0.000857455 | up |
| hsa-miR-410-3p | -3.205355341 | 0.010359787 | down |
| hsa-miR-381-3p | -3.138865914 | 2.87E-06 | down |
| hsa-miR-205-5p | -3.073945764 | 0.01422305 | down |
| hsa-miR-873-5p | -3.005962644 | 0.032200642 | down |
| hsa-miR-627-5p | 2.99767298 | 0.038565997 | up |
| hsa-miR-203a-3p | -2.972559399 | 0.001665987 | down |
| hsa-miR-132-5p | -2.741924186 | 0.000360328 | down |
| hsa-miR-184 | -2.681685053 | 0.000245392 | down |
| hsa-miR-708-3p | -2.665719404 | 0.001383923 | down |
| hsa-miR-324-3p | 2.536861855 | 0.000476337 | up |
| …… |  |  |  |

**SUPPLEMENTARY TABLE 1** Top 30 differential miRNAs between the severe acne patients and the health controls.
